# Challenging management of severe chronic disorders in acute pandemic situation: Chronic liver disease under COVID-19 pandemic as the proof-of-principle model to orchestrate the measures in 3PM context

**DOI:** 10.1007/s13167-021-00231-8

**Published:** 2021-03-03

**Authors:** Lubomir Skladany, Tomas Koller, Svetlana Adamcova Selcanova, Janka Vnencakova, Daniela Jancekova, Viktoria Durajova, Lukas Laffers, Juraj Svac, Katarina Janickova, Michal Palkovič, Pavel Kohout, Olga Golubnitschaja

**Affiliations:** 1 HEGITO (Div. Hepatology, Gastroenterology, and Liver Transplantation) of the Department of Internal Medicine II, Faculty of Medicine, Slovak Medical University, F. D. Roosevelt Teaching Hospital, Banska Bystrica, Slovakia; 2 5th Department of Internal Medicine, Comenius University Faculty of Medicine, University Hospital Bratislava Ruzinov, Bratislava, Slovakia; 3 Department of Science and Research, F.D. Roosevelt Teaching Hospital, Banska Bystrica, Slovakia; 4 Department of Mathematics, Faculty of Natural Sciences, Matej Bel University, Banska Bystrica, Slovakia; 5 Central Evidence Department, Health Care Surveillance Authority (HCSA), Bratislava, Slovakia; 6 Forensic Medicine and Pathological Anatomy Department, Health Care Surveillance Authority (HCSA), Bratislava, Slovakia; 7 Department of Internal Medicine, 3Rd Medical Faculty Charles University, Thomayer Hospital Prague, Prague, Czech Republic; 8 Predictive, Preventive Personalised (3P) Medicine, Department of Radiation Oncology, University Hospital Bonn, Rheinische Friedrich-Wilhelms-Universität Bonn, Bonn, Germany; 9 3PM Research Unit, Department of Radiation Oncology, University Hospital, Medical Faculty, Rheinische Friedrich-Wilhelms-Universität Bonn, 53107 Bonn, Germany

**Keywords:** Predictive preventive personalized medicine (PPPM/3PM), COVID-19, SARS CoV-2, Pandemic, Acute situation, Tertiary care, Priority pathways, Chronic disorder, Liver disease, Acute liver failure, Hepatitis, Live transplantation, Advanced stages, Cirrhosis registry, Death rates, Disease management, Optimal healthcare modeling, Patient needs, Statistics, Electronic patient records, Personalized treatment algorithms, Expert recommendations, Health policy

## Abstract

Chronic liver disease management is a comprehensive approach requiring multi-professional expertise and well-orchestrated healthcare measures thoroughly organized by responsible medical units. Contextually, the corresponding multi-faceted chain of healthcare events is likely to be severely disturbed or even temporarily broken under the force majeure conditions such as global pandemics. Consequently, the chronic liver disease is highly representative for the management of any severe chronic disorder under lasting pandemics with unprecedented numbers of acutely diseased persons who, together with the chronically sick patient cohorts, have to be treated using the given capacity of healthcare systems with their limited resources. Current study aimed at exploring potentially negative impacts of the SARS CoV-2 outbreak on the quality of the advanced chronic liver disease (ACLD) management considering two well-classified parameters, namely, (1) the continuity of the patient registrations and (2) the level of mortality rates, comparing pre-COVID-19 statistics with these under the current pandemic in Slovak Republic. Altogether 1091 registrations to cirrhosis registry (with 60.8% versus 39.2% males to females ratio) were included with a median age of 57 years for patients under consideration. Already within the very first 3 months of the pandemic outbreak in Slovakia (lockdown declared from March 16, 2020, until May 20, 2020), the continuity of the patient registrations has been broken followed by significantly increased ACLD-related death rates. During this period of time, the total number of new registrations decreased by about 60% (15 registrations in 2020 *versus* 38 in 2018 and 38 in 2019). Corresponding mortality increased by about 52% (23 deaths in 2020 *versus* 10 in 2018 and 12 in 2019). Based on these results and in line with the framework of 3PM guidelines, the *pandemic priority pathways* (PPP) are strongly recommended for maintaining tertiary care uninterrupted. For the evidence-based implementation of PPP, creation of predictive algorithms and individualized care strategy tailored to the patient is essential. Resulting classification of measures is summarized as follows:

- The *Green PPP Line* is reserved for prioritized (urgent and comprehensive) treatment of patients at highest risk to die from ACLD (tertiary care) as compared to the risk from possible COVID-19 infection.
- The *Orange PPP Line* considers patients at middle risk of adverse outcomes from ACLD with re-addressing them to the secondary care. As further deterioration of ACLD is still probable, pro-active management is ascertained with tertiary center serving as the 24/7 telemedicine consultation hub for a secondary facility (on a physician-physician level).
- The *Red PPP Line* is related to the patients at low risk to die from ACLD, re-addressing them to the primary care. Since patients with stable chronic liver diseases without advanced fibrosis are at trivial inherent risk, they should be kept out of the healthcare setting as far as possible by the telemedical (patient-nurse or patient- physician) measurements.

The assigned priority has to be monitored and re-evaluated individually—in intervals based on the baseline prognostic score such as MELD. The approach is conform with principles of predictive, preventive and personalized medicine (PPPM / 3PM) and demonstrates a potential of great clinical utility for an optimal management of any severe chronic disorder (cardiovascular, neurological and cancer) under lasting pandemics.

## Introduction

### Acute pandemic condition is challenging for the entire healthcare community

The outbreak of a severe acute respiratory syndrome coronavirus 2 (SARS-CoV-2) infection classified by the WHO on March 11, 2020, as a pandemic, is currently hitting again and will ultimately affect more than half of the world’s population [1–8]. The first wave mortality had varied from 0.2 to 4% with outliers such as Italy, where the case fatality reached 12% [1–4]. Pandemic measures, lockdowns, restructuring, and adaptation of hospital capacities, and widespread cancelations of planned healthcare have resulted in a secondary impact on all patients due to record-high waiting times and significantly fewer patients being diagnosed with common cardiovascular or metabolic diseases [9]. In hardly hit areas, even urgent healthcare such as cancer therapy has been affected, due to preemptive canceling or due to an overwhelmed system of healthcare [10].

During the provisional “cease-fire” following the first wave, medical professionals were preparing for the next hit by counting and sorting casualties while trying to understand the pandemic behavior on their battlefields. Challenges brought by the pandemic situation confronted physicians with unprecedented numbers of patients who were managed by a very limited number of human and material resources. The circumstances may invite for a more widespread consideration for medicine that is based on predictive, preventive, and personalized tools. The approach may be generalized to the entire population encouraging government authorities for using individual behavior data from a myriad of devices and social networks [11] to trace contacts and to monitor outbreak dynamics in real time [12]. Also, several COVID-19 disease predictive models have been recently proposed, while newer models based on the unique character of the immune response are emerging [11, 13]. Finally, in the context of specialized care, disease-specific prognostic models play an integral part in the disease management and are ready for being evaluated as tools of predictive, preventive, and personalized approaches during pandemic times.

### Pandemic impact on the management of advanced chronic liver disease

It has been shown that SARS-CoV-2 causes mild liver injury in nearly half of the previously healthy people. However, in 1 to 11% of patients with pre-existing liver cirrhosis, COVID-19 increases mortality and skews pathways of cirrhosis care [4, 14–24]. Slovakia is repeatedly ranking top five in Europe in cirrhosis mortality, which is the new leading cause of death in the age group between 25 and 45 years [25–27]. The first wave of COVID-19 pandemic hit the Slovak healthcare system in the process of a continually increasing number of resource-intensive patients with advanced chronic liver disease (ACLD) gravitating to a gradually decreasing number of specialized, government-operated, tertiary centers. Very important factor which needs to be taken into account when analyzing the interaction of the COVID-19 outbreak with Slovak healthcare system’s limited capacity is the immediate and intensive government response after the occurrence of the first COVID-19 case together with almost absolute public compliance with the anti-pandemic measures [28].

Lockdown in Slovakia was declared by the Slovak Government on March 16, 2020, and lasted with the highest intensity until May 20, 2020. In general, it was characterized by social distancing, movement restrictions, a universal obligation for wearing facemasks, closing of all schools, government offices, non-essential commerce, and workplaces while canceling all public or family gatherings. The negative impact of lockdown on cirrhosis management could be summarized as follows*: *(1) virtually all outpatient visits were changed from personal contact to telemedicine. The exceptions were early postoperative and unstable liver transplant (LT) patients [29, 30]. Outpatient care was very limited for patients with resistant ascites (telemedicine, adjusting diuretic doses by phone, evading taps). (2) In the hospital setting, liver unit beds were transformed into the hospital COVID-19 area. There were two substantial differences in the management of patients with acute decompensation (AD) of ACLD including acute-on-chronic liver failure (ACLF): (2A) a substantial elevation of the threshold for AD admission to hospital compared with the pre-COVID-19 period, and (2B) a delimitation of admissions to lower-rank facilities according to the district. These two measures have remarkably diminished tertiary care for AD ACLD. (3) Our liver unit has ceased the experimental therapies for non-responders with severe alcohol-associated hepatitis and practically canceled any tertiary care for these patients. (4) Liver transplantations were suspended for all the indications except for acute liver failure.

## Patients and methods

### Advanced chronic liver disease registry under COVID-19 condition

The Department of Hepatology, Gastroenterology, and Liver Transplantation (HEGITO) of F.D. Roosevelt Teaching Hospital, Banska Bystrica, Slovakia, provides tertiary referral services for the catchment area of 650 000 inhabitants and ultimate (national) referral services for LT. Since 2014, we have been enrolling patients admitted with ACLD who provided written informed consent into the ACLD registry named Registry HEGITO 7 (RH7) [31]. Here, we aimed to make use of a unique opportunity provided by the RH7 registry to observe the impact of the COVID-19 outbreak on registry operation and the outcome of ACLD patients. We have selected two well-classified parameters with the highest potential for capturing pandemic damage and its mode of action, these being (1) the continuity of the patient registrations and (2) the level of mortality rates, comparing pre-COVID-19 statistics with these under pandemic in Slovak Republic. We hypothesized fewer registrations and higher pandemic-associated mortality and subordinated our methodology to the goal of discerning possible disparities in temporal trends.

### Identifying unmet needs and potential solutions in the context of 3P medicine

We have attempted to translate results to a concrete predictive, preventive, and personalized medicine (3PM/PPPM) guideline as presented and widely encouraged in PPPM-dedicated publications by the European Association for Predictive, Preventive, and Personalized Medicine (EPMA) [32, 33]. Such guidance could serve as the proof-of-principle model and could potentially be extrapolated as a blueprint for the 3PM approach in the management of other chronic conditions during upcoming pandemic waves.

### Statistical methods and predictive models

We have diagnosed ACLD by the standard criteria and recorded demographics, laboratory, and imaging findings necessary to describe ACLD by etiology, type, and degree of decompensation (e.g., AD, chronic [CD], [ACLF]), as well as decompensating events and outcome including mortality. Death-related data for the Registry cohort were obtained from the Health Care Surveillance Authority of the Slovak Republic on a regular weekly basis from two registries: (1) national database of the insured inhabitants, date of death; and (2) registry of deaths of individuals declared dead, specifically adjusted for recording COVID-19 as the main cause of death.

**Fig. 1:**
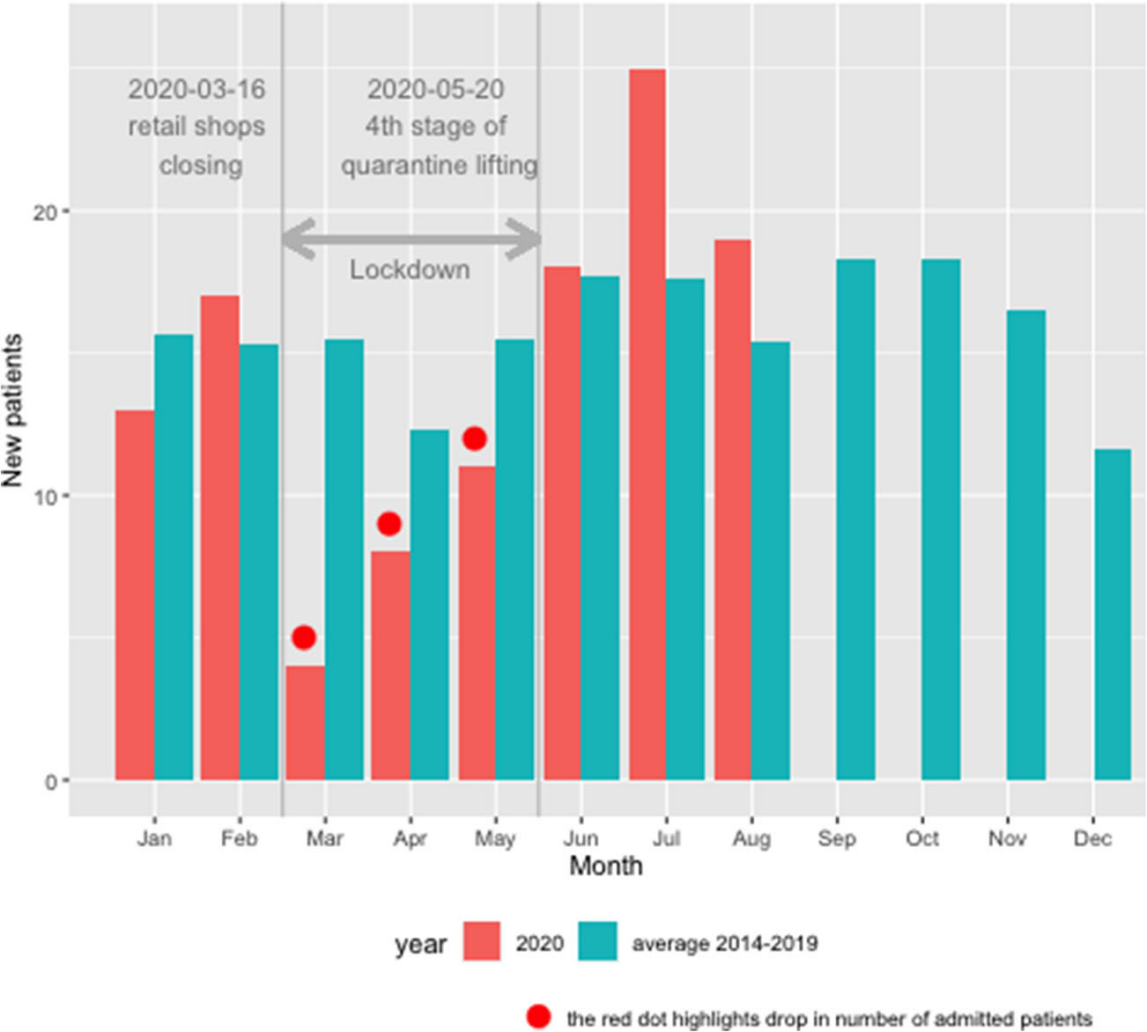
Monthly numbers of newly admitted patients. Monthly numbers of new registrations to ACLD registry RH-7, a decline in new registrations during the first three months of the quarantine (March, April, and May 2020 [red dots]). We associate this decline (average drop during these three months is aprox. 53%) with anti-pandemic measures which kept patients with liver cirrhosis out of tertiary liver care

The registry cohort was divided into two groups according to its immediate experience: patients who passed away before March 16, 2020, formed the pre-COVID-19 group, and those alive on March 16, 2020, were included in the COVID-19 group. Primary endpoints are the rate of new registrations and short-term mortality (Fig. 1). Having 1091 patients in the cirrhosis RH7 registry admitted before March 16, 2020, we conducted a survival analysis using a **Cox proportional hazard model** using the R environment for statistical computing with the package “survival”. [34–36]. The following factors were used: gender, age, body mass index (BMI [kg/m^2^]), Child-Turcotte-Pugh score (CTPS), model for end-stage liver disease (MELD), liver frailty index (LFI), ACLF, triceps skinfold fat (TSF [cm]), dynamometry (hand-grip strength [HGS, kg]), C-reactive protein (CRP), and leukocytes (Leu). We used this model to predict individual median residual lifetime for 563 patients (median age 55.7, MELD 14, 57.2% male) alive on March 16, with 95% confidence intervals. We compared the actual cumulative number of deaths in the sample between March 16 and August 31 with predictions based on the Cox proportional hazard model [34–36]. Since the median-based survival prediction curve is likely to underestimate survival at the beginning of the study period, we conducted a similar analysis for the two preceding years (2019 and 2018) serving as a useful control group.

## Results

### Impact of COVID-19 outbreak on the continuity of patient registrations

In total, we analyzed 1091 registrations (60.8% males/39.2% females), with a median age of 57 years and a MELD score of 16. In the pre-pandemic years, we enrolled approximately 156 patients per year, while ACLD was caused mostly by alcohol-associated liver disease (ALD). The baseline characteristics of the study sample are depicted in Table 1.

**Table 1:** Baseline characteristics of advanced chronic liver disease patients in the RH7 registry, March 16, 2020 (*N* = 1 091)

Variable	Median/count	Range/proportion
Age	57	19–83
Females	427	29.14%
Males	664	60.86%
Etiology of ACLD:		
Alcoholic liver disease	751	68.84%
Autoimmune (AIH, PBC, PSC)	109	9.99%
Viral (HBV, HCV)	102	9.35%
Other	147	13.47%
Unknown	84	7.70%
Scoring systems:		
MELD score	16	6–88
Child-Pugh-Turcotte score	9	5–20
Liver frailty index	3.35	1.69–6.84
Mortality		
1-year mortality	430	39.37%
Yearly registrations		
2014	70	6.42%
2015	165	15.12%
2016	191	17.51%
2017	151	13.84%
2018	224	20.53%
2019	256	23.46%
2020 (as of March 16th, 2020)	34	10.54%
	*N* = 1000*	
Chronic decompensation	529	52,9%
Acute decompensation	398	39,8%
	*N* = 1091**	
ACLF 1	56	5.13%
ACLF 2	41	3.75%
ACLF 3	25	2.29%

*ACLD* advanced chronic liver disease (cirrhosis); *ALD *alcohol associated liver disease; *HBV* hepatitis B virus; *HCV*  hepatitis C virus; *AIH* autoimmune hepatitis; *PBC* primary biliary cholangitis; *PSC* primary sclerosing cholangitis; *MELD *model for end-stage liver disease; *CTPS* Child-Turcotte-Pugh score; *ACLF* acute on chronic liver failure; *LFI* liver frailty index

^*^Electronic and paper files of these 1000 patients were double-checked per protocol by one investigator (D.J.)

^**^Data on remaining 91 patients are straight registry uploads

**Fig. 2:**
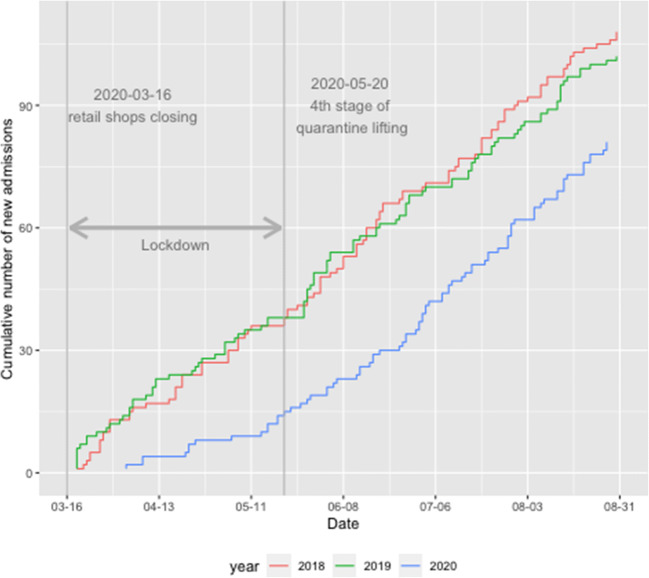
New admissions since March 16, 2020. New hospital admissions, comparison of the corresponding time frame between years pre-COVID-19 (red: 2018, green: 2019) and COVID-19 (blue: 2020). During the spring of 2020 quarantine, significantly fewer patients (approx. 60%) were admitted in comparison with two previous years 2018 and 2019 (green and red lines)

During the first 3 months of the quarantine (March, April, and May 2020) we observe a sharp drop (approx. by 47% in comparison to average across years 2014–2019) in the number of the new registrations of the cirrhosis patients in our RH7 registry as a reflection of the anti-pandemic measures declared by state authorities (Fig. 1). The following months then show a steep increase in registrations while reaching and overshooting the pre-lockdown numbers in June, July, and August 2020, respectively. In Fig. 2, we display the rate of new admissions to the hospital during the COVID-19 period (from March 16 to August 31) compared with the two previous years. The sharp decline during the COVID-19 period is apparent, while the rate of new hospital admissions starts to increase after May 20, 2020. Since this date, the slope of the curves does not differ among all 3 years.

### Impact of COVID-19 outbreak on all-cause mortality

**Fig. 3:**
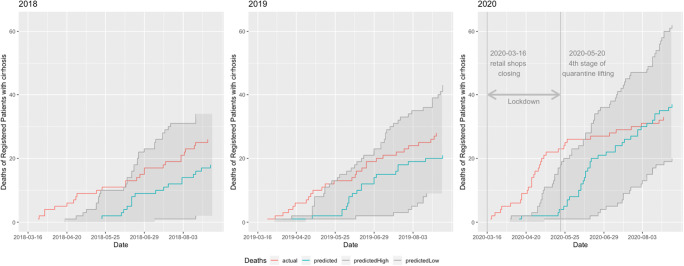
Actual deaths versus median predicted deaths. All-cause mortality in the ACLD registry RH7. The lines display actual deaths *versus* median predicted deaths. Actual deaths in 2018 and 2019 (left and middle panes) were within the 95% confidence interval. In the spring of 2020, the number of actual deaths sharply increased and was above the numbers predicted by Cox proportional hazard model, showing excess mortality (19 excess deaths in 2020 in comparison to 10 in 2018 and 10 in 2019) indicating the negative secondary impact of initial pandemic measures on tertiary cirrhosis care

We compared the cumulative incidence of median predicted deaths with the actual deaths. In Fig. 3, it is apparent that while actual deaths in our predictive models for expected death in cirrhosis patients in 2018 and 2019 (left and middle panel) were well within the 95% confidence intervals, in 2020 (the right panel), during the COVID-19 outbreak and consecutive quarantine period, the number of actual deaths of our cirrhosis patients sharply increased (approx. by 52%, 23 deaths in 2020 in comparison to 10 in 2018 and 12 in 2019) and was well above predicted numbers. Shortly before the last stage of quarantine, as depicted by a gray vertical line, the curve noticeably flattens.

## Discussion

Our study provides new evidence that the COVID-19 outbreak in our healthcare setting resulted in excess mortality of the particular patients suffering from advanced chronic liver disease. Increased mortality was causally related to a decrease in tertiary hospital admissions for decompensated ACLD, as captured by fewer new registrations to the cirrhosis registry. This chain of causation would imply two important lessons we have learned: (1) the number of new registrations to our cirrhosis registry could be perceived as the surrogate marker for tertiary liver care, and (2) the specialized cirrhosis care in our healthcare context plays an irreplaceable role.

### COVID-19 and tertiary ACLD care

COVID-19 pandemic has impacted cirrhotic patients twice: the first time directly by SARS-CoV-2 infection and the second time indirectly by the consequences of distorted liver care [20, 29, 37–40]. Reports are showing that patients with cirrhosis are at increased risk of SARS-CoV-2 infection and mortality, both directly and indirectly mediated by COVID-19 [19, 20, 24, 41–44]. Evidence is pointing to increased mortality of hospitalized patients with cirrhosis when compared to pre-pandemic time, increased in-hospital mortality of cirrhotic patients with COVID-19 as compared to patients with COVID-19 without cirrhosis, and increased mortality of patients with cirrhosis and COVID-19 as compared to patients with cirrhosis without COVID-19 [15, 18, 21, 24, 42, 44–46]. Patients themselves might also have suffered more anxiety which could be associated with risky behavior including alcohol intake [47–52]. During the spring of 2020 outbreak, our obvious priority was to protect ACLD patients from the risk of contracting then-unknown SARS-CoV-2 infection [19, 53]. Here, we provide *important supporting evidence for the real-life effectiveness of the stringent lockdown measures* as we recorded zero COVID-19 deaths in our registry [54, 55].

Reports have been published from other cirrhosis registries operating during the COVID-19 pandemic, such as Secure Cirrhosis Registry, COVID-Hep.net, COVID Cirrhosis.org, and COVID-Cirrhosis-CHESS. In brief, they have shown a relationship between SARS-CoV-2 infection, stage of cirrhosis (MELD > 14, CTPS C), liver decompensation, and mortality [18, 19, 44, 56]. Our findings in relation to the ones mentioned provide evidence of the secondary impact of the COVID-19 outbreak on specialized care, particularly in the setting of ACLD in a country with high cirrhosis burden. Our observation serves as an indicator of the influence of global unprecedented (read: “not amenable to proper preparedness”) pandemic measures on death rates unrelated to the very cause of the pandemic; leading us to the urgent need for a new approach towards similar situations in the future. Our results also lend support to the case raised by Tapper et al. in the field of hepatology and by other authors from different specialties that distorted specialized care during lockdown could have serious adverse consequences on health-related outcomes. When aiming to prevent increased mortality during the expected future outbreaks, various authors issued a call for keeping the specialized care uninterrupted [20, 29, 52, 54, 55, 57–59]. As the next waves of the pandemic are expected to hit countries that were relatively spared during the first wave (i.e., Slovakia, Greece, or Malta), we have to be prepared and try to translate evidence collected during the initial outbreak to a new approach [7] using the practical and well-evidenced predictive, preventive, and personalized approach.

## Expert recommendations in the context of 3P medicine

The new challenge lies in guaranteeing the best possible trade-off between the benefits of maintaining high-quality medical care on the one hand and the risk of nosocomial exposure to SARS-CoV-2 infection on the other one [19, 20, 37]. So far, it has been shown that the risk of nosocomial transmission of SARS-CoV-2 varied between 9,9 and 40% with aerosolization procedures such as gastrointestinal endoscopy being at the right side of the spectrum and that the reported rate of SARS-CoV-2-induced acute liver injury was 50% [4, 21, 60–64]. To counterbalance the risk from SARS-CoV-2 inherent in keeping liver care uninterrupted, hepatology-specific preventive measures protecting patients and caregivers from infection have been proposed [5, 19, 62, 65–71]. Our results have clearly shown that a safety liver hub reserved for SARS-CoV-2-negative ACLD patients is not an option in our region, since it would necessitate their delimitation to secondary liver care with consequently increased mortality. We interpret these results as a very strong indicator of the pivotal role the tertiary liver care plays in our healthcare setting.

### Pandemic priority pathways suggested

**Fig. 4:**
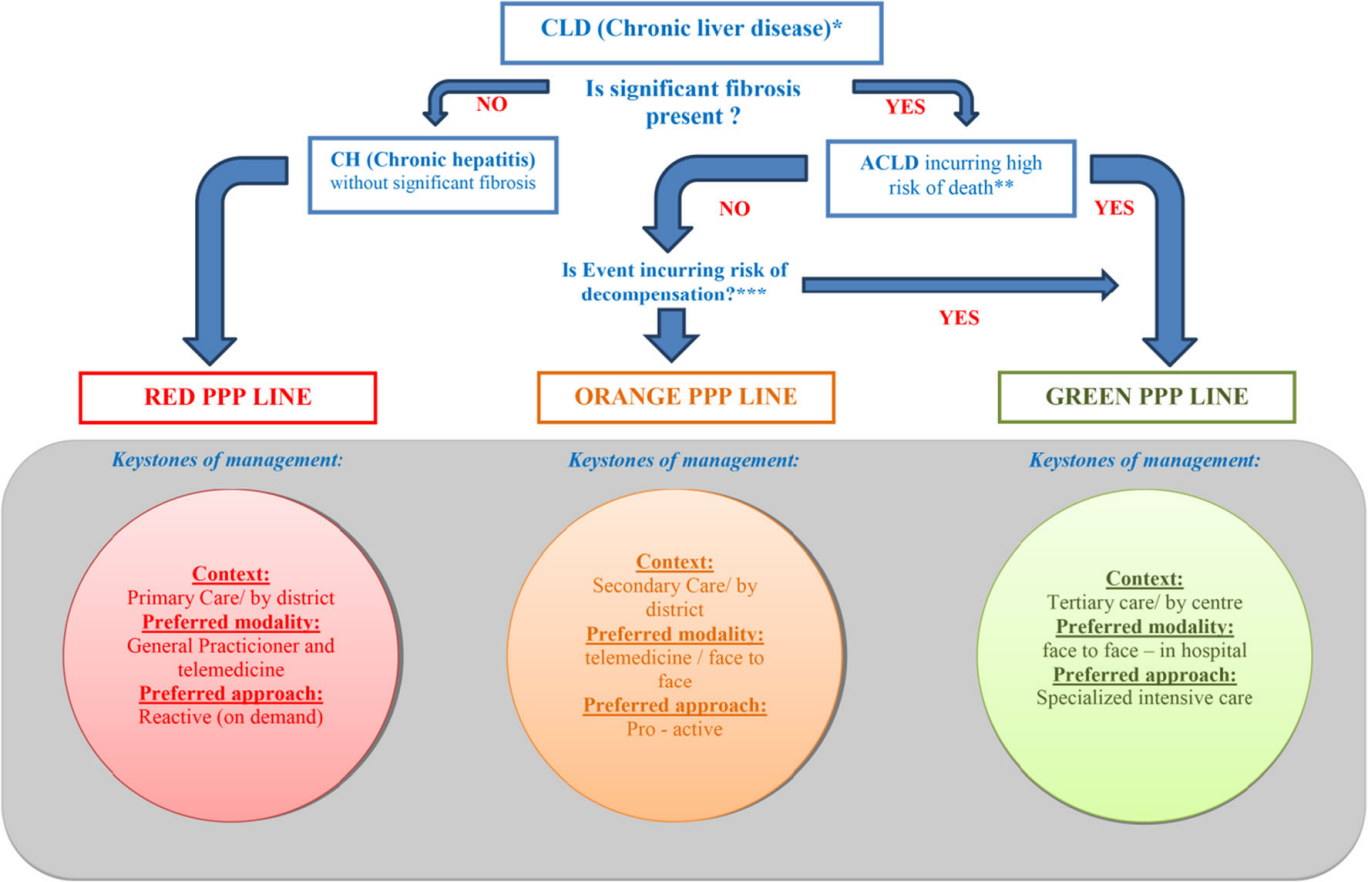
Pandemic priority pathways (PPP): Triage of patients with chronic liver disease on a new clinical event (Event)—as suggested by our results showing excess mortality in delayed tertiary cirrhosis care. Traffic light style pandemic priority pathways (PPP) personalized for patients with the advanced chronic liver disease based on predictive models aiming to prevent adverse health-related outcomes. **Red** PPP Line (= “STOP” (in primary care)) for patients with chronic hepatitis without significant fibrosis of any etiology. Reactive (on demand): visit by telemedicine only when the condition deteriorates. **Orange** PPP Line (= “PREPARE” (for tertiary care while under secondary care)) Proactive monitoring: MELD (model for end-stage liver disease) score < 14, regular pre-scheduled visits, on-demand consultation when changed sleep or mood pattern; asterixis; fever, oliguria; anuria; hematemesis; melena; jaundice; ascites. **Green** PPP Line (= “GO!” (to tertiary care)) will be reserved for patients with generic syndromes: liver transplantation (LT) candidates, MELD > 14, acute on chronic liver failure (ACLF), acute decompensation of ACLD (advanced chronic liver disease), acute liver faliure (ALI), severe alcoholic hepatitis (SAH) not responding to steroids, hepatocellular carcinoma (HCC)

For this reason, we are suggesting a **predictive**,** preventive**, and** personalized** approach offering to our unstable patients with ACLD both: specialized liver care and protection from COVID-19. Having this aim in mind, we have developed an institutional liver-specific **pandemic priority pathways (PPP)**. At the personal level, patients in our cirrhosis registry as well as all the newly consulted patients will be assigned a traffic light priority color based on their degree of **predicted risk of death from ACLD** (Fig. 4, Table 2). Our liver unit will issue identification cards in a traffic colors style which will link each patient to its PPP and assist them with passing hospital anti-pandemic barriers and shortening door–to–care time. Risk stratification and PPP color assignment will be reassessed at intervals based on the prognostic ACLD score (the MELD score, model for end-stage liver disease) reflecting a 3-month probability of death. Our predictive recommendations have been based on several lines of evidence [5, 14, 15, 19, 72–78]. Namely, in Europe’s most COVID-19 affected Italy, Ivarone et al. have demonstrated that CLIF-SOFA score in acute decompensation (AD), and MELD > 15 in chronic decompensation (CD) to be the independent predictors of death [21, 42, 79, 80]. These findings were consistent with Moon et al. showing that another prognostic score, the Child–Pugh-Turcotte score was associated with a 12–63% excess of liver-related mortality in the registry as compared to 79% lung-related and 4,3% cardiac-related mortality [44].

**Table 2:** Definition of the patient group assigned to the GREEN PPP line, patients in need of preserved tertiary care

GREEN pandemic priority pathway (ppp) = go directly to tertiary care Do not delay/deviate tertiary care for the following groups of ACLD patients
1. Registered in the cirrhosis registry (RH7)
2. Not registered, but share similar characteristics (see inclusion criteria to RH7)
3. Acute-on-chronic liver failure (ACLF) or first acute decompensation of any subtype, especially if indicated to liver transplantation (LT)
4. Candidates for LT, especially if the indication is a. urgent (acute liver failure) [82, 83], or b. ACLF (although not yet formally accepted as urgent in Slovakia) [84], or c. Severe acute alcoholic hepatitis (SAH) within Lille criteria [85, 86]
5. Severe alcoholic hepatitis particularly non-responders to, or not eligible for, corticosteroid therapy
6. Indicated for secondary treatment modalities for chronic decompensation, e.g., - Transjugular portosystemic shunt (TIPSS) or permanent drainage for refractory ascites - Closure of the shunt (TIPSS) in persistent hepatic encephalopathy (HE) - Need for locoregional therapy for HCC

*ACLD* advanced chronic liver disease; *ACLF* acute in chronic liver failure; *HCC* hepatocellular carcinoma; *HE* hepatic encephalopathy; *LT *liver transplantation; *PPP* pandemic priority pathway; *RH7* Registry HEGITO (Div. hepatology, gastroenterology and liver transplantation) 7; *TIPS* transjugular intrahepatic portosystemic shunt

**Fig. 5:**
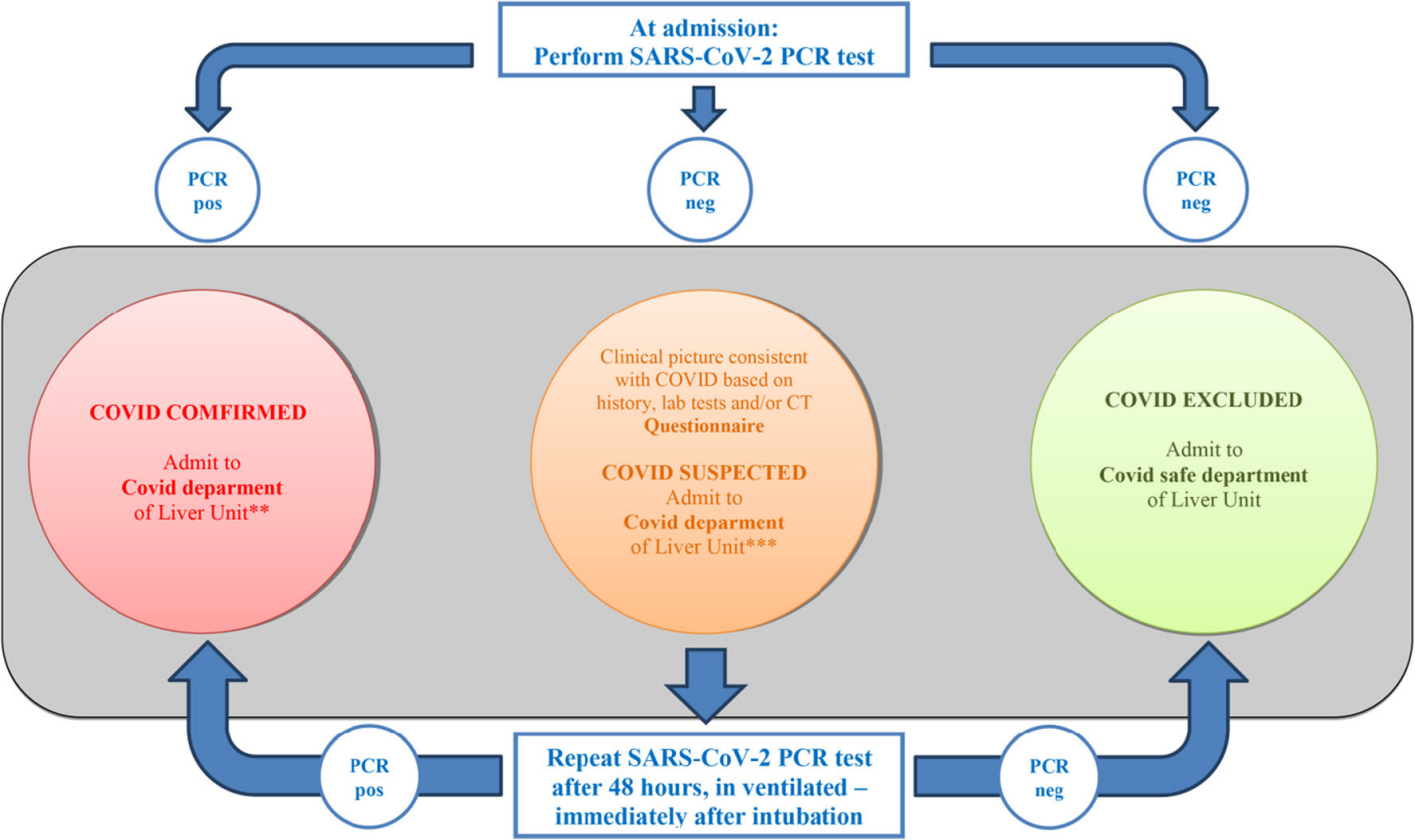
Recall policy in patients with ACLD* (Advanced chronic liver disease) allocated in Green PPP (Pandemic priority pathways) line with management preferred in tertiary care. Traffic light style recall policy for patients with advanced chronic liver disease allocated to the Green PPP line with management being preferred in tertiary care. Patients fast-tracked to tertiary cirrhosis care should be safeguarded from nosocomial infection with SARS-CoV-2 based on PCR test performed on admission (SARS-CoV-2: Severe acute respiratory syndrome–Coronavirus 2; PCR: polymerase chain reaction test)

Green PPP Line (= “GO!” (to tertiary care)) will be reserved for patients in whom the risk from ACLD far outweighs the risk from possible COVID-19 [81], and should lead patients straight to tertiary care. The main generic syndromes which should be passed quickly via the Green PPP Line are acute liver failure, decompensated cirrhosis with additional characteristics inflicting an increased risk of adverse outcomes—such as a new AD, any acute-on-chronic liver failure (ACLF), previous CD, severe alcoholic hepatitis (SAH), or liver transplantation (LT, patients or candidates) (Table 2). To qualify our facility for the Green PPP Line of unimpeded liver care, it is of uppermost importance to simultaneously safeguard our incoming patients from the risk of SARS-CoV-2 infection [20, 37]. For this purpose, we elaborated the Green PPP Line to the explicit details such as arrows and signs, dedicated doors, lifts, rooms, and caregivers (Table 3). Patients moving along the Green PPP Line will be subjected to a standardized COVID-19 triage policy (Fig. 5. Importantly, we will have to communicate and pre-conceive this strategy with patients as well as with our referring counterparts from secondary care. At the same time, we are preparing communication lines for telemedicine (physician-physician; patient-physician, [11].

**Table 3:** Four-step plan preparing the institution for providing personalized tertiary cirrhosis care during SARS—CoV-2 (Severe acute respiratory syndrome–Coronavirus 2) outbreak

Predictive, preventive, and personalized medical approach proposal for cirrhosis care during the pandemic
1. *Prepare your liver care in advance:* a. Acknowledge your position in the context of cirrhosis care: i Secondary care 1. District hospital with internal medicine beds, ICU, and consultant in gastroenterology/hepatology on duty or call 2. Locate your proxy tertiary referral center, assure contact lines (names of consultants, telephone numbers, e-mail addresses, etc.) 3. Prepare telemedicine on the physician – physician level ii. Tertiary care 1. Regional, university/academic hospital with the liver unit and specialized ICU. Liver transplant unit–ultimate referral center 2. Locate your partners from lower-rank facilities, be at their disposal 3. Prepare telemedicine (lines, e-mails, etc.) physician-to-physician, and patient (from registry)-to-physician iii. Based on your own data analysis, or the basis of the recommendations from similar-to-your healthcare environments and *act in advance*
2. *Evaluate the personalized risk* of adverse outcomes in your cirrhosis patients, based on the clinical scenario and disease predictive scores a. In the registry (tertiary center) b. On admission (secondary center)
3. *Allocate personal PPP* accordingly (issue [print] personal PPP Card with traffic light color before possible Event [Fig. 4])
4. *Allocate personal COVID- 19 protection module* (on Event [Fig. 5])

*ICU* intensive care unit; *PPP* pandemic priority pathway

Orange PPP Line (= “PREPARE” (for tertiary care while under secondary care)) concerns patients in whom the risk of a bad outcome from cirrhosis is trivial and would keep them at the level of secondary care. However, everything would be prepared for a fast transfer to a tertiary facility in case things went wrong: close monitoring and pro-active management of specific complications of cirrhosis are ascertained, and the telemedicine is exercised on the physician-physician level (secondary care-tertiary care). The tertiary center would serve as the consultation hub. Patients kept on the Orange PPP Line are those with ACLD without prior decompensation, with decompensating events not incurring the high risk of death such as trivial infections with sufficient and fast response to therapy, minor traumas, slowly evolving ascites, peri-malleolar edemas, but also some high-risk patients, if allocated to palliative care for being too sick or non-compliant/not eligible for third-line treatments (Table 4).

**Table 4.** Definition of the patient group assigned to the ORANGE PPP line, not requiring immediate tertiary care during the SARS-CoV 2 (Severe acute respiratory syndrome–Coronavirus 2) outbreak

Orange pandemic priority pathway (PPP) = “PREPARE” [for tertiary care in secondary care]
1. ACLD without prior decompensation (events not incurring the high risk of death) - Trivial infections with sufficient and fast response to therapy - Minor traumas - Slowly evolving ascites (Grades I–II) - Peri-malleolar (Grade I) edemas
2. High-risk patients allocated to palliative care - Too sick (disseminated malignancies, too sick to be transplanted due to cardiac, pulmonary, nutrition, frailty, infections, etc. issues) - Non-compliance (persistent alcohol abuse, etc.) - Not eligible for second-, or third-line treatments, such as transjugular intrahepatic portosystemic shunt, extracorporeal modalities (plasmapheresis, hemodialysis, other), experimental therapy for patients with severe alcohol-associated alcoholic hepatitis not responding to corticosteroids (Lille-model liver transplant, fecal microbial transplantation, growth factors, other), etc.

*ACLD* advanced chronic liver disease; *PPP*  pandemic priority pathway

Red PPP Line (= “STOP” (in primary care)) concerns patients whose immediate risk from liver disease is negligible. This PPP line would stop patients in primary care or the realm of telemedicine and should be reserved for patients with chronic hepatitis without significant fibrosis with the possible exceptions for autoimmune hepatitis not responding to corticosteroids, new Wilson’s disease, and few other syndromes (Table 5) [29].

**Table 5:** Definition of the patient group assigned to stay in primary care during the SARS-CoV 2 (Severe acute respiratory syndrome–Coronavirus 2) outbreak

Red pandemic priority pathway (PPP) = “STOP” [in primary care]
Chronic hepatitis without significant fibrosis (stable diseases): - Autoimmune hepatitis on treatment - Alcohol-related liver disease - Chronic hepatitis B - Chronic hepatitis C - Hereditary hemochromatosis - Non-alcoholic fatty liver disease (NAFLD) of metabolic dysfunction-associated liver disease (MAFLD) - Primary biliary cholangitis - Primary sclerosing cholangitis - Other
Exceptions: - Acute flare of chronic hepatitis B - Autoimmune hepatitis not responding to corticosteroids - New Wilson’s disease - Primary sclerosing cholangitis with recurrent bouts of bacterial cholangitis

### Strengths and limitations

Our study provides a rather unique reflection of the impact of lockdown on the conserved core processes inside the cirrhosis registry (fewer registrations) and their consequences (increased mortality). We consider our cirrhosis registry with its 6-year history of patient enrollment and standardized data collection an appropriate tool for reliable reflection of temporal dynamics. To the best of our knowledge, RH7 can also be considered an unparalleled instrument in the context of central European countries. Since we were not able to fetch detailed information on the circumstances of death from other institutions and authorities (e.g., whether patients have passed away at home or in hospital), our results cannot be directly compared to those provided by other authors [43]. However, they can complement existing knowledge by providing evidence that the lockdown measures are very effective in terms of new SARS-CoV-2 infection but are associated with increased mortality in decompensated cirrhosis without COVID-19 [43]. Although we base our conclusions and recommendations on a regional dataset, they are well adaptable to specific conditions of other countries—taking, of course, into consideration the local and even institutional healthcare setting and pandemic countermeasures.

## Conclusion and recommendations

Our study adds to the existing evidence on the high anti-SARS-CoV-2 effectivity of lockdown measures by real-life data. The findings complement current knowledge by showing that patients with decompensated ACLD are at significant risk of health-threatening consequences of the currently applied anti-pandemic measures. COVID-19-unrelated mortality has increased in our cirrhosis registry which is likely related to the temporary interruption in tertiary liver care. With the new evidence in hand and the imminent pandemic waves in the context of the practical application of predictive, preventive, and personalized medicine approach, we suggest to consider and to follow “pandemic priority pathways”, which may help to decrease the excess mortality in ACLD. The proposed approach selects a personalized PPP line of care, based on predictive models, therefore maintaining uninterrupted tertiary care for patients with a high risk of adverse outcomes. Green PPP Line (= “GO!” (to tertiary care)) will be reserved for patients for whom the health risks from ACLD far outweighs the risks from possible COVID-19 infection and would lead them straight to tertiary care. Orange PPP Line (= “PREPARE” (for tertiary care while under secondary care)) concerns patients for whom the risk of adverse outcome of cirrhosis is rather limited and would keep them at the level of secondary care. Red PPP Line (= “STOP” (in primary care)) concerns patients whose immediate risks from liver disease is negligible. This PPP line would keep the patients in primary care or re-address them to the telemedical management. The assigned lines would be subjected to the periodical re-assessment based on the actual prognostic score. The proposed strategy requires specialized disease management to be applied synergistically with an effective COVID-19 triage and prevention policy. There is a potential of the presented strategy to serve as a blueprint for an optimal management of other chronic diseases if “pandemic priority pathways” will get adjusted to specific requirements in corresponding countries and to specific patient cohorts.

## Abbreviations

ACLD: Advanced chronic liver disease
ACLF: Acute on chronic liver failure
AD: Acute decompensation
AIH: Autoimmune hepatitis
ALD: Alcohol-associated liver disease
BMI: Body mass index
CD: Chronic decompensation
CLIF—SOFA score: Chronic liver failure—the sequential organ failure assessment score
COVID: Coronavirus disease 19
CRP: C-reactive protein
CTPS: Child-Turcotte-Pugh score
EPMA: European Association for Predictive, Preventive, and Personalized Medicine
HBV: Hepatitis B virus
HCC: Hepatocellular carcinoma
HCV: Hepatitis C virus
HEGITO: Div. Hepatology, Gastroenterology, and Liver Transplantation
HGS: Handgrip strength
LFI: Liver frailty index
LEU: Leukocytes
LT: Liver transplantation
MELD: Model for end-stage liver disease
PBC: Primary biliary cholangitis
PPP: Pandemic priority pathways
PPPM: Preventive, predictive, and personalized medicine
PSC: Primary sclerosing cholangitis
RH7: Registry HEGITO 7
SAH: Severe alcoholic hepatitis
SARS-CoV-2: Severe acute respiratory syndrome coronavirus 2
TIPS: Transjugular intrahepatic portosystemic shunt
TSF: Triceps skinfold
WHO: World Health Organization
